# 4-(Carboxy­meth­yl)anilinium chloride

**DOI:** 10.1107/S1600536809021849

**Published:** 2009-06-13

**Authors:** Habiba Kechout, Ratiba Belhouas, Sofiane Bouacida, Hocine Merazig

**Affiliations:** a Unité de Recherche de Chimie de l’Environnement et Moléculaire Structurale, CHEMS, Faculté des Sciences Exactes, Département de Chimie, Université Mentouri Constantine, 25000 Algeria; b Faculté de Chimie, USTHB, BP32 El-Alia, Bab-Ezzouar, Alger, Algeria

## Abstract

In the crystal of the title compound, C_8_H_10_NO_2_^+^·Cl^−^, alternating layers of hydro­phobic and hydro­philic zones stack along the *c* axis. The chloride anions are sandwiched between the 4-(carboxy­meth­yl)anilinium layers, forming inter­molecular O—H⋯Cl and N—H⋯Cl hydrogen bonds with the ammonium and carboxyl groups of the cations. In addition, inter­molecular N—H⋯O and weak C—H⋯O and C—H⋯Cl hydrogen bonds help stabilize the crystal structure.

## Related literature

For our ongoing studies of hydrogen-bonding inter­actions in the crystal structures of protonated amines, see: Benslimane *et al.* (2007 ▶); Bouacida *et al.* (2005*a* ▶,*b* ▶,*c* ▶, 2006 ▶, 2007 ▶, 2008 ▶, 2009 ▶). For amino acids in which the amino N atom is protonated, see: Bouacida *et al. *(2006); Rademeyer (2004*a* ▶,*b* ▶). For a related structure, see: Benslimane *et al.* (2007 ▶). For bond-length data, see: Allen *et al.* (1987 ▶).
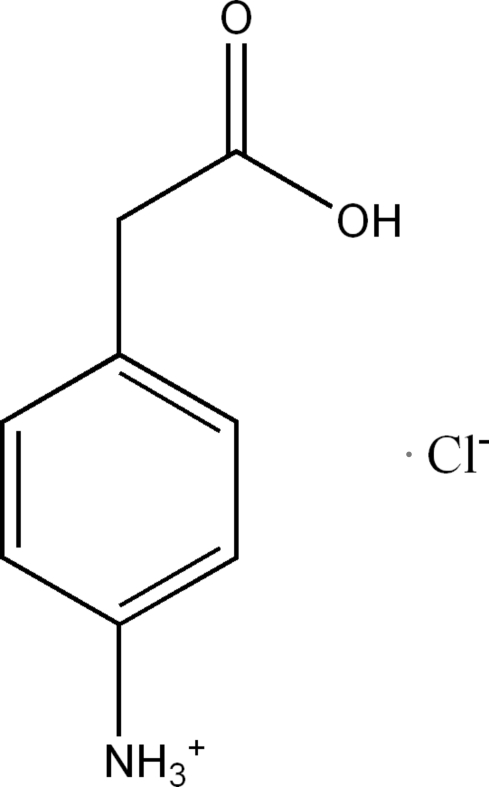


## Experimental

### 

#### Crystal data

C_8_H_10_NO_2_^+^·Cl^−^
*M* *_r_* = 187.62
Monoclinic, 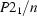

*a* = 4.4982 (4) Å
*b* = 11.0790 (11) Å
*c* = 17.7120 (17) Å
β = 95.429 (3)°
*V* = 878.73 (14) Å^3^
*Z* = 4
Mo *K*α radiation
μ = 0.39 mm^−1^
*T* = 100 K
0.44 × 0.12 × 0.1 mm

#### Data collection

Bruker APEXII diffractometer
Absorption correction: multi-scan (*SADABS*; Bruker, 1998 ▶) *T*_min_ = 0.809, *T*_max_ = 0.962
7536 measured reflections
2006 independent reflections
1785 reflections with *I* > 2σ(*I*)
*R*_int_ = 0.040

#### Refinement

*R*[*F* ^2^ > 2σ(*F* ^2^)] = 0.033
*wR*(*F* ^2^) = 0.08
*S* = 1.03
2006 reflections
113 parameters
H-atom parameters constrained
Δρ_max_ = 0.30 e Å^−3^
Δρ_min_ = −0.22 e Å^−3^

### 

Data collection: *APEX2* (Bruker, 2001 ▶); cell refinement: *SAINT* (Bruker, 2001 ▶); data reduction: *SAINT*; program(s) used to solve structure: *SIR2002* (Burla *et al.*, 2005 ▶); program(s) used to refine structure: *SHELXL97* (Sheldrick, 2008 ▶); molecular graphics: *ORTEP-3 for Windows* (Farrugia, 1997 ▶) and *DIAMOND* (Brandenburg & Berndt, 2001 ▶); software used to prepare material for publication: *WinGX* (Farrugia, 1999 ▶).

## Supplementary Material

Crystal structure: contains datablocks global, I. DOI: 10.1107/S1600536809021849/lh2838sup1.cif

Structure factors: contains datablocks I. DOI: 10.1107/S1600536809021849/lh2838Isup2.hkl

Additional supplementary materials:  crystallographic information; 3D view; checkCIF report

## Figures and Tables

**Table 1:** Hydrogen-bond geometry (Å, °)

*D*—H⋯*A*	*D*—H	H⋯*A*	*D*⋯*A*	*D*—H⋯*A*
O1—H1⋯Cl1^i^	0.82	2.20	3.0087 (13)	171
N1—H1*A*⋯O2^ii^	0.89	1.98	2.8517 (17)	167
N1—H1*B*⋯Cl1^iii^	0.89	2.41	3.2285 (13)	152
N1—H1*C*⋯Cl1^iv^	0.89	2.26	3.1516 (14)	174
C2—H2⋯O2^ii^	0.93	2.49	3.2338 (18)	137
C3—H3⋯Cl1	0.93	2.82	3.7481 (15)	175

Symmetry codes: (i) 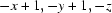; (ii) 
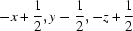
; (iii) 
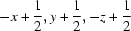
; (iv) 
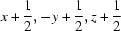
.

**Fig. 1.**
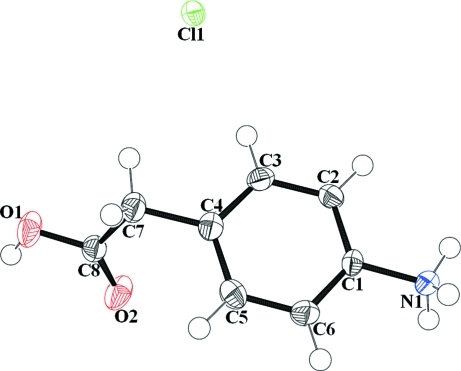
The asymmetric unit of the title compound with the atomic labelling scheme. Displacement are drawn at the 50% probability level.

**Fig. 2.**
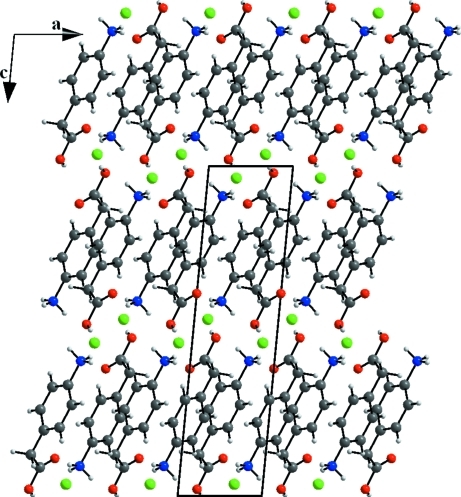
Part of the crystal structure illustrating the molecular layers, viewed along the b axis.

**Fig. 3.**
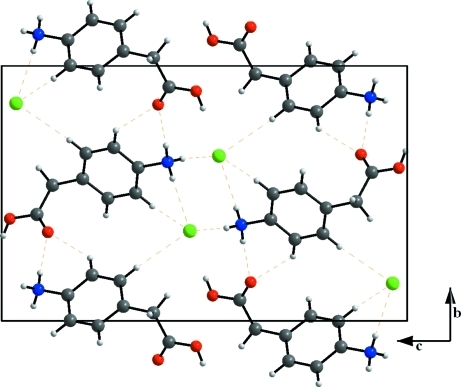
Part of the crystal structure with hydrogen bonds shown as dashed lines, viewed along the a axis.

## supplementary crystallographic information

### Comment

The title compound, was prepared as part of our ongoing studies of hydrogen-bonding interactions in the crystal structure of protonated amines (Bouacida *et al.*, 2005*a*,*b*,*c*, 2006, 2007, 2008, 2009).

The molecular structure of (I), and the atomic numbering used, is illustrated in Fig. 1. All bond distances (Allen *et al.*, 1987) and angles are within the ranges of accepted values. The amino N atom is protonated as in previously reported amino acids (Bouacida & al., 2006; Rademeyer, 2004*a*,*b*). The layered crystal packing of (I) is shown in Fig. 2, in which cations form alterning layers of 4-(carboxymethyl)anilinium of hydrophobic and hydrophilic zones along the *c* axis, and the chloride ions are located between these layers. In the structure, two types of classical hydrogen bonds are observed, *viz*. cation–anion and cation–cation (Fig. 3). The 4-(carboxymethyl)anilinium cations and the chloride anions form hydrogen-bonded double layers at *z* = 0 and *z* = 1/2, linked by N—H···Cl, C—H···Cl and O—H···Cl hydrogen bonds. Additional hydrogen-bonding parameters are listed in Table 1. These interaction bonds link the cations and the anions together, forming a three-dimensional network and reinforcing the cohesion of the ionic structure.

### Experimental

The title compound was crystallized by slow evaporation of an aqueous solution of 4-aminophenyl acetic acid, tin(II) chloride dihydrate and hydrochloric acid in a molar ratio of 5:5:1. White stick-like crystals were obtained after two weeks.

### Refinement

All H atoms were located in Fourier maps but introduced in calculated positions and treated as riding on their parent C, O and N atoms with C—H = 0.93–0.97 Å, O—H = 0.82Å and N—H = 0.89Å and *U*_iso_(H) =1.5–1.2(carrier atom).

### Crystal data

**Table:**

C_8_H_10_NO_2_^+^·Cl^−^	*F*(000) = 392
*M**_r_* = 187.62	*D*_x_ = 1.418 Mg m^−^^3^
Monoclinic, *P*2_1_/*n*	Mo *K*α radiation, λ = 0.71073 Å
Hall symbol: -P 2yn	Cell parameters from 3208 reflections
*a* = 4.4982 (4) Å	θ = 2.3–27.4°
*b* = 11.0790 (11) Å	µ = 0.39 mm^−^^1^
*c* = 17.7120 (17) Å	*T* = 100 K
β = 95.429 (3)°	Stick, white
*V* = 878.73 (14) Å^3^	0.44 × 0.12 × 0.1 mm
*Z* = 4	

### Data collection

**Table:**

Bruker APEXII diffractometer	1785 reflections with *I* > 2σ(*I*)
graphite	*R*_int_ = 0.040
CCD rotation images, thin slices scans	θ_max_ = 27.5°, θ_min_ = 3.7°
Absorption correction: multi-scan (*SADABS*; Bruker, 1998)	*h* = −5→5
*T*_min_ = 0.809, *T*_max_ = 0.962	*k* = −14→14
7536 measured reflections	*l* = −22→22
2006 independent reflections	

### Refinement

**Table:**

Refinement on *F*^2^	Primary atom site location: structure-invariant direct methods
Least-squares matrix: full	Secondary atom site location: difference Fourier map
*R*[*F*^2^ > 2σ(*F*^2^)] = 0.033	Hydrogen site location: inferred from neighbouring sites
*wR*(*F*^2^) = 0.08	H-atom parameters constrained
*S* = 1.03	*w* = 1/[σ^2^(*F*_o_^2^) + (0.0283*P*)^2^ + 0.5301*P*] where *P* = (*F*_o_^2^ + 2*F*_c_^2^)/3
2006 reflections	(Δ/σ)_max_ = 0.001
113 parameters	Δρ_max_ = 0.30 e Å^−^^3^
0 restraints	Δρ_min_ = −0.22 e Å^−^^3^

### Special details

**Table:**

Geometry. All e.s.d.'s (except the e.s.d. in the dihedral angle between two l.s. planes) are estimated using the full covariance matrix. The cell e.s.d.'s are taken into account individually in the estimation of e.s.d.'s in distances, angles and torsion angles; correlations between e.s.d.'s in cell parameters are only used when they are defined by crystal symmetry. An approximate (isotropic) treatment of cell e.s.d.'s is used for estimating e.s.d.'s involving l.s. planes.
Refinement. Refinement of *F*^2^ against ALL reflections. The weighted *R*-factor *wR* and goodness of fit *S* are based on *F*^2^, conventional *R*-factors *R* are based on *F*, with *F* set to zero for negative *F*^2^. The threshold expression of *F*^2^ > σ(*F*^2^) is used only for calculating *R*-factors(gt) *etc*. and is not relevant to the choice of reflections for refinement. *R*-factors based on *F*^2^ are statistically about twice as large as those based on *F*, and *R*- factors based on ALL data will be even larger.

### Fractional atomic coordinates and isotropic or equivalent isotropic displacement parameters (Å^2^)

**Table:**

	*x*	*y*	*z*	*U*_iso_*/*U*_eq_	
Cl1	0.33999 (7)	0.14718 (3)	0.035821 (18)	0.01567 (11)	
O1	0.7756 (3)	0.59938 (11)	0.02054 (6)	0.0270 (3)	
H1	0.7238	0.6660	0.0040	0.040*	
O2	0.5016 (3)	0.65210 (10)	0.11399 (6)	0.0257 (3)	
N1	0.3016 (3)	0.37706 (11)	0.40929 (7)	0.0145 (3)	
H1A	0.1824	0.3128	0.4051	0.022*	
H1B	0.1969	0.4414	0.4212	0.022*	
H1C	0.4494	0.3642	0.4455	0.022*	
C1	0.4271 (3)	0.39836 (13)	0.33671 (8)	0.0126 (3)	
C2	0.3601 (3)	0.32006 (13)	0.27659 (8)	0.0148 (3)	
H2	0.2377	0.2535	0.2817	0.018*	
C3	0.4804 (3)	0.34312 (13)	0.20801 (8)	0.0155 (3)	
H3	0.4373	0.2911	0.1672	0.019*	
C4	0.6631 (3)	0.44257 (13)	0.19984 (8)	0.0145 (3)	
C5	0.7279 (3)	0.51913 (14)	0.26215 (8)	0.0176 (3)	
H5	0.8524	0.5853	0.2576	0.021*	
C6	0.6098 (3)	0.49813 (14)	0.33049 (8)	0.0166 (3)	
H6	0.6522	0.5499	0.3714	0.020*	
C7	0.7903 (3)	0.46817 (14)	0.12557 (8)	0.0180 (3)	
H7A	0.7456	0.4006	0.0916	0.022*	
H7B	1.0060	0.4744	0.1347	0.022*	
C8	0.6709 (3)	0.58249 (14)	0.08711 (8)	0.0157 (3)	

### Atomic displacement parameters (Å^2^)

**Table:**

	*U*^11^	*U*^22^	*U*^33^	*U*^12^	*U*^13^	*U*^23^
Cl1	0.01972 (19)	0.01379 (19)	0.01353 (18)	0.00041 (13)	0.00178 (13)	0.00186 (12)
O1	0.0392 (7)	0.0206 (6)	0.0239 (6)	0.0128 (5)	0.0181 (5)	0.0103 (5)
O2	0.0361 (7)	0.0226 (6)	0.0203 (6)	0.0151 (5)	0.0123 (5)	0.0061 (5)
N1	0.0169 (6)	0.0146 (6)	0.0124 (6)	−0.0011 (5)	0.0034 (5)	−0.0013 (5)
C4	0.0141 (6)	0.0146 (7)	0.0150 (7)	0.0054 (5)	0.0026 (5)	0.0034 (5)
C1	0.0131 (6)	0.0142 (7)	0.0108 (6)	0.0020 (5)	0.0025 (5)	0.0023 (5)
C6	0.0173 (7)	0.0163 (7)	0.0160 (7)	−0.0025 (6)	0.0003 (5)	−0.0027 (6)
C3	0.0190 (7)	0.0138 (7)	0.0137 (7)	0.0024 (6)	0.0011 (5)	−0.0020 (5)
C7	0.0199 (7)	0.0173 (7)	0.0178 (7)	0.0043 (6)	0.0071 (6)	0.0034 (6)
C8	0.0156 (7)	0.0161 (7)	0.0157 (7)	0.0003 (6)	0.0034 (5)	0.0007 (6)
C5	0.0152 (7)	0.0173 (7)	0.0206 (7)	−0.0039 (6)	0.0032 (5)	0.0013 (6)
C2	0.0161 (6)	0.0123 (7)	0.0159 (7)	−0.0021 (6)	0.0014 (5)	0.0000 (6)

### Geometric parameters (Å, °)

**Table:**

O1—C8	1.3234 (17)	C1—C6	1.388 (2)
O1—H1	0.8200	C6—C5	1.387 (2)
O2—C8	1.2121 (18)	C6—H6	0.9300
N1—C1	1.4709 (17)	C3—C2	1.399 (2)
N1—H1A	0.8900	C3—H3	0.9300
N1—H1B	0.8900	C7—C8	1.512 (2)
N1—H1C	0.8900	C7—H7A	0.9700
C4—C3	1.390 (2)	C7—H7B	0.9700
C4—C5	1.401 (2)	C5—H5	0.9300
C4—C7	1.5100 (19)	C2—H2	0.9300
C1—C2	1.384 (2)		
			
C8—O1—H1	109.5	C4—C3—H3	119.5
C1—N1—H1A	109.5	C2—C3—H3	119.5
C1—N1—H1B	109.5	C4—C7—C8	113.72 (12)
H1A—N1—H1B	109.5	C4—C7—H7A	108.8
C1—N1—H1C	109.5	C8—C7—H7A	108.8
H1A—N1—H1C	109.5	C4—C7—H7B	108.8
H1B—N1—H1C	109.5	C8—C7—H7B	108.8
C3—C4—C5	118.62 (13)	H7A—C7—H7B	107.7
C3—C4—C7	121.06 (13)	O2—C8—O1	123.32 (14)
C5—C4—C7	120.33 (13)	O2—C8—C7	124.41 (13)
C2—C1—C6	121.69 (13)	O1—C8—C7	112.27 (12)
C2—C1—N1	119.89 (12)	C6—C5—C4	121.20 (14)
C6—C1—N1	118.42 (12)	C6—C5—H5	119.4
C5—C6—C1	118.77 (13)	C4—C5—H5	119.4
C5—C6—H6	120.6	C1—C2—C3	118.67 (13)
C1—C6—H6	120.6	C1—C2—H2	120.7
C4—C3—C2	121.05 (13)	C3—C2—H2	120.7
			
C2—C1—C6—C5	−0.1 (2)	C4—C7—C8—O1	−176.97 (13)
N1—C1—C6—C5	−179.72 (13)	C1—C6—C5—C4	0.7 (2)
C5—C4—C3—C2	0.7 (2)	C3—C4—C5—C6	−1.0 (2)
C7—C4—C3—C2	−179.38 (13)	C7—C4—C5—C6	179.07 (13)
C3—C4—C7—C8	113.71 (16)	C6—C1—C2—C3	−0.2 (2)
C5—C4—C7—C8	−66.37 (18)	N1—C1—C2—C3	179.42 (12)
C4—C7—C8—O2	3.9 (2)	C4—C3—C2—C1	−0.1 (2)

### Hydrogen-bond geometry (Å, °)

**Table:**

*D*—H···*A*	*D*—H	H···*A*	*D*···*A*	*D*—H···*A*
O1—H1···Cl1^i^	0.82	2.20	3.0087 (13)	171
N1—H1A···O2^ii^	0.89	1.98	2.8517 (17)	167
N1—H1B···Cl1^iii^	0.89	2.41	3.2285 (13)	152
N1—H1C···Cl1^iv^	0.89	2.26	3.1516 (14)	174
C2—H2···O2^ii^	0.93	2.49	3.2338 (18)	137
C3—H3···Cl1	0.93	2.82	3.7481 (15)	175

Symmetry codes: (i) −*x*+1, −*y*+1, −*z*; (ii) −*x*+1/2, *y*−1/2, −*z*+1/2; (iii) −*x*+1/2, *y*+1/2, −*z*+1/2; (iv) *x*+1/2, −*y*+1/2, *z*+1/2.
